# Parental and Peer Relationships and Their Impact on Symptom Severity in Adolescent Patients With Anorexia Nervosa

**DOI:** 10.1002/erv.70072

**Published:** 2025-12-27

**Authors:** Armita Tschitsaz, Andrea M. Schumacher, Stefan Lerch, Andrea Wyssen, Marialuisa Cavelti, Ines Mürner‐Lavanchy, Julian Koenig, Michael Kaess, Franziska Schlensog‐Schuster

**Affiliations:** ^1^ University Hospital of Child and Adolescent Psychiatry and Psychotherapy and Psychotherapy University of Bern Bern Switzerland; ^2^ Division of Youth Mental Health, Department of Psychology University of Basel Basel Switzerland; ^3^ Department of Child and Adolescent Psychiatry, Psychosomatics and Psychotherapy, Faculty of Medicine and University Hospital Cologne University of Cologne Cologne Germany; ^4^ Department of Child and Adolescent Psychiatry Centre for Psychosocial Medicine University Hospital Heidelberg Heidelberg Germany; ^5^ Department of Child and Adolescent Psychiatry, Psychotherapy and Psychosomatics University of Leipzig Leipzig Germany

**Keywords:** anorexia nervosa, adolescence, bullying, eating disorder, maternal relationship, parental discrepancy, peer victimisation, perpetration

## Abstract

**Objective:**

Perceived parental relationship characteristics, such as maternal overprotection, rejection or neglect, and peer victimisation, are suggested to be more common in patients with anorexia nervosa (AN) than in healthy controls. This study compares parental and peer relationships in adolescent patients with AN to those in a clinical control group (CC; a matched group of patients with other mental disorders) and investigates their association with AN severity.

**Method:**

Self‐reported parental and peer relationships were compared between adolescents with AN (*n* = 43) and CC (*n* = 127), matched for age, sex and global functioning. Multiple linear and logistic regression were used to analyse the association between parental and peer relationships and AN severity.

**Results:**

The AN group exhibited a more positive overall evaluation of parental relationships (*b* = 18.34, *p* = 0.002), particularly with fathers (*b* = 5.30, *p* = 0.028), fewer parental discrepancies (*b* = −7.67, *p* = 0.013), less peer victimisation (OR = 0.43, *p* = 0.030) and perpetration (OR = 0.26, *p* = 0.006) than the CC group. No significant associations were identified between these relationships and AN severity.

**Conclusions:**

In contrast to previous studies comparing social relationships in patients with AN and healthy controls, our findings suggest that increased positive parental and peer relationships may serve as a resource, irrespective of symptom severity.

## Highlights

- Adolescents with anorexia nervosa (AN) reported more positive parental relationships, particularly with their fathers, and experienced less peer victimisation and perpetration compared to clinical controls (CC).
- Despite differences in parental and peer relationships between AN and CC groups, these factors were not significantly associated with the severity of AN symptoms.
- Unlike previous studies comparing AN patients with healthy controls, our findings raise the question of whether positive parental and peer relationships serve as a resource for adolescents with AN, independent of symptom severity.

## 1 Introduction

The incidence, prevalence and symptom severity of anorexia nervosa (AN) in adolescents has increased significantly in recent years (Herpertz‐Dahlmann 2024), particularly among those in the prepubertal stage or at the onset of puberty (van Eeden et al. 2023). Etiological models of AN emphasise the complex interplay of biological, psychological, behavioural, psychosocial and interactional factors that contribute to the development and maintenance of this disorder. It is well documented that interpersonal challenges such as critical family ruptures, bullying, social exclusion and stressful social relationships are common risk factors for the development and maintenance of AN (Treasure et al. 2020). Moreover, patients with AN appear to experience impairments in both self‐ and interpersonal functioning (Schumacher et al. 2024), which in turn may influence relationship quality. In their cognitive‐interpersonal model of AN, Treasure et al. (2020) point to evidence that specific problems in social cognition and emotion regulation (e.g., social anxiety symptoms), may have long‐term effects on the patient's interpersonal environment, which in turn reinforces illness severity.

Previous research shows that in comparison to healthy controls, women across eating disorder (ED) diagnostic categories report lower parental care together with higher parental protection (Tetley et al. 2014). This seemingly paradoxical relationship style has also been found to be more prevalent in individuals with AN compared to non‐clinical controls (Tetley et al. 2014). Studies focussing on AN describe positive associations between increased maternal control and symptom severity, while paternal care is negatively associated with symptom severity in young adults with AN compared to healthy controls (Canetti et al. 2008). Vidović et al. (2005) found that young adults with ED, including AN, reported a lack of cohesion, flexibility as well as impaired communication with their mothers in comparison to healthy young adults. More recently, studies found that parental interpersonal style appears to be less caring, disengaged, poorly interwoven, rigid, less cohesive and less communicative (Tafà et al. 2017; Treasure et al. 2020). Gruber et al. (2020), using the same parental questionnaire as in the present study, found that adolescents and young adults with AN and bulimia nervosa reported significantly higher levels of maternal overprotection as well as rejection and neglect compared to healthy individuals. In contrast, paternal relationships did not differ significantly.

Studies examining differences between patients with AN compared to other psychiatric disorders are limited and less consistent in terms of experienced parental relationships. Tetley et al. (2014) reported that most studies found little difference in parental bonding in women with ED compared to women with other psychiatric disorders. This supports the assumption that parental relationship quality could be a general risk factor in the development or maintenance of AN (Del Casale et al. 2023). However, few studies have compared patients with AN and borderline personality disorder, they found that the AN group received higher levels of parental care (comparable to healthy controls) than the borderline personality disorder group (Laporte and Guttman 2007; Lee and Vaillancourt 2024; Tetley et al. 2014). It remains unclear whether the higher level of care in AN is specific to comparisons with borderline personality disorder or extendable to other mental health conditions. Furthermore, ED diagnoses are categorised inconsistently in the studies. This underscores the need to analyse family relationships in the context of AN and other clinical populations to refine and adapt the interpersonal and systemic aspects of AN therapy.

In an effort to gain insight into the relationship between family dynamics and ED, research has proposed a number of explanations, such as patients' avoidant problem‐solving styles or denial (Tetley et al. 2014; Vidović et al. 2005), an insecure attachment style, and specific parental behaviours like those related to care and control, which have proven to reduce identity development, self‐acceptance (Gander et al. 2015; Izydorczyk et al. 2021), the capacity of self‐regulation (Vidović et al. 2005) and responsibility for eating behaviour (Gruber et al. 2020). Examining varying perspectives on relationship quality offers additional insight into the causes of relationship difficulties. Thus, Dancyger et al. (2005) found that adolescents with different ED diagnoses tended to rate their maternal relationships more negatively than their mothers, while adolescents and their parents did not differ in their ratings of depressive symptoms. Similarly, Gruber et al. (2020) reported that relationships with mothers were perceived as more conflictual than relationships with fathers. These results highlight the importance that patients with AN receive an assessment of their relationship quality with each parent individually. Ciao et al. (2015) showed that reported improvements in family relationship were associated with better family‐based treatment outcomes for all family members. Conversely, other studies have demonstrated that significant symptom improvements in adolescents with AN are associated with better functioning in family relationship (Balottin et al. 2018; Wallis et al. 2017). These improvements were observed both in dyadic relationships with mother and father and in triadic relationships between parents and patient, underscoring that relational skills within the family are associated with a positive prognosis in adolescent patients with AN.

Beyond family relations, impairments in peer relationships and specific forms of social aggression such as bullying, have been identified as a significant risk factor for the development of a wide range of psychological problems, including low self‐esteem, ED symptoms and negative body image (Seiffert et al. 2024; Thornberg et al. 2020). A meta‐analysis by Lie et al. (2019) found that patients with ED and CC were significantly more likely to be bullied and teased compared to healthy controls. Specifically, 17% of patients with ED, 17% of CC and 10% of healthy controls reported past experiences of bullying. Both, patients with AN and those with other ED showed a positive correlation between bullying and anorexic severity at the trend level. With regard to bullying perpetration, individuals with AN and bulimia nervosa were less likely to engage in perpetration compared to both healthy and psychiatric controls. Therefore, bullying victimisation appears to be particularly relevant to the development and severity of ED (Copeland et al. 2015; Lie et al. 2019; Westwood et al. 2016), as social relationships become more important during adolescence and may have a significant impact on adolescents' self‐image and experiences of mastery (Copeland et al. 2015; Day et al. 2022).

Overall, a bidirectional association between relationship qualities and symptom severity in AN is likely. A recent study by Lukas et al. (2022) demonstrated that adolescents with AN experience poorer relationship quality not only with their parents, but also with their peers compared to healthy controls. Amongst patients, negative correlations were identified between parental or peer relationships and anorexic pathology. These correlations were attributed to higher levels of alexithymia, which may influence the quality of relationships and, in turn, exacerbate anorexic symptoms. In addition, Gruber et al. (2020) found that harm prevention was a partial mediator between overprotection in parental relationship and anorexic symptoms. However, there are few studies with mixed methodological approaches that have comprehensively investigated both associations between parental relationships (assessing the relationship to the mother and the father individually) and peer relationships in patients with AN (Colla et al. 2023; Lukas et al. 2022; Yamamiya and Stice 2024). While Lukas et al. (2022) and Yamamiya and Stice (2024) found positive correlations between the quality of parental and peer relationships, Colla et al. (2023) highlighted both as key psychosocial resources. However, none of the authors drew conclusions regarding the directionality of these associations. A developmental meta‐analysis by Schulz et al. (2023) demonstrated that cross‐sectional analyses revealed bidirectional associations between the quality of parental and peer relationships. Moreover, the cited longitudinal studies have identified mutually supportive parent‐child relationships as a significant foundation for the quality of subsequent relationships. Consequently, bullying may be regarded as a risk factor, while positive parental relationships may function not only as a protective factor but also as a fundamental resource for coping with episodic relational difficulties, such as those with peers.

This study aims to examine the association between parental and peer relationships in terms of bullying in patients with AN compared to CC. It is hypothesised that patients diagnosed with AN report less difficulties in their relationships with their parents compared to CC (hypothesis 1, H1). Additionally, potential differences in the quality of maternal versus paternal relationships are examined for patients with AN and CC. Regarding peer problems, it is hypothesised that individuals with AN report a lower prevalence of bullying victimisation and perpetration compared to CC (hypothesis 2, H2). Among patients with AN, a negative association between parental relationship and symptom severity (hypothesis 3, H3) as well as a positive association between victimisation, perpetration and symptom severity is expected (hypothesis 4, H4). Finally, it is hypothesised that parental relationship quality serves as a protective factor for the association between bullying and symptom severity among adolescents with AN (hypothesis 5, H5).

## 2 Method

### 2.1 Participants and Procedures

The current study used a shared data set repository (Schumacher et al. 2024), which pooled data from two separate studies: the Anorexia Registry Study (Herpertz‐Dahlmann and Hebebrand 2017), which commenced in July 2021 and is ongoing, and the Bern Basic Documentation (BeBaDoc), which was initiated in November 2018 and ended in December 2022. This analysis includes data collected from both studies up to October 2022. Participants were recruited from general psychiatric inpatient and day‐care units, from inpatient‐equivalent home treatment (BeBaDoc study) and a specialised therapy centre for EDs (Anorexia Registry Study) at the University Hospital of Child and Adolescent Psychiatry and Psychotherapy, Switzerland.

Inclusion criteria for both studies were adolescents between 11 and 18 years old with sufficient German language skills. Exclusion criteria were problems understanding study details or not providing informed consent. The inclusion criteria for the Anorexia Registry Study included the additional requirement of an AN diagnosis as assessed by the Mini International Neuropsychiatric Interview for Children and Adolescents (MINI‐KID). Participants of the CC group did not meet the diagnostic criteria for AN according to the MINI‐KID. Written informed consent was obtained from all participants, and also from parents for those under 14 years of age. After admission to the clinic, assessments were carried out by well‐trained PhD or undergraduate psychology students. The studies were conducted in accordance with the Declaration of Helsinki (World Medical Association 2013) and approved by the local ethics committees (BeBaDoc Ethics ID: 2018‐01339; and Anorexia Registry Ethics ID: 2021‐00234).

### 2.2 Measures

*Demographic Data*: Demographic information was collected using a standardised set of questions to assess age, sex and educational level.

#### 2.2.1 Assessments of the BeBaDoc Study, Completed by AN and CC

##### 2.2.1.1 The Mini International Neuropsychiatric Interview for Children and Adolescents (MINI‐KID)

Assessment of current psychiatric disorders according to DSM‐IV and ICD‐10 was based on the MINI‐KID (Sheehan et al. 2010), which is a structured interview that allows valid and reliable psychiatric diagnoses for children and adolescents (Duncan et al. 2018).

##### 2.2.1.2 Children's Global Assessment Scale (CGAS)

A single‐item instrument that evaluates psychological and social overall level of functioning on a scale of 1–100, with high scores indicating low functional impairment (Shaffer et al. 1983). Good psychometric properties have been published with high reliability scores as well as discriminant and concurrent validity (Shaffer et al. 1983).

##### 2.2.1.3 Parental Representation Screening Questionnaire (PRSQ)

The child version of the PRSQ (Titze et al. 2010) was used to assess the parent‐child relationship from the perspective of adolescents. The PRSQ comprises 36 items per parent, which are divided into three resource scales (cohesion, identification, autonomy), five risk scales (conflict, punishment, rejection/indifference, emotional boundary overstepping, anxiety/overprotection) and the additional help scale, which measures the extent to which the child or adolescent feels that parents are supportive or helpful in various areas of life. The first 34 items for each parent are scored on a five‐point frequency scale, while the last two items (identification scale) are scored on a five‐point agreement scale. As described in the manual, four index scores were calculated to assess the adolescent's perception of the parental relationship: the mother relationship quality (BQM), the father relationship quality (BQF), their parental discrepancy (i.e., the difference in perception between the two relationships), and the overall relationship quality (i.e., the difference between the sum of the maternal and paternal relationship scores and the discrepancy between the two parents' scores). The strengths of the PRSQ lies in its ability to assess the perceived relationship with both parents separately, and to compare their responses to standardised reference groups, which consider age and gender differences in perception (Titze et al. 2010). Psychometric qualities in adolescents have been reported with internal consistencies above 0.80 in 12 of the 16 PRSQ scales (Titze et al. 2010). The internal consistency (Cronbach's α) of the total score of the present study was 0.81, of the relationship quality to the mother 0.77 and to the father 0.74.

##### 2.2.1.4 Forms of Bullying Scale (FBS)

Experiences of bullying were assessed using the two subscales of victimisation and perpetration (Shaw et al. 2013). Both scales comprise 10 items, each assessing five distinct types of bullying through two questions: Each item is rated on a five‐point Likert scale (1 = ‘This did not happen to me’, 2 = ‘Once or twice’, 3 = ‘Every few weeks’, 4 = ‘About once a week’, 5 = ‘Several times a week or more’). In this study, the total score was computed and classified into no bullying (sum score of up to 2 = ‘once or twice’), occasional bullying (sum score of 3 = ‘every few weeks’), or frequent bullying (sum score of at least 4 = ‘once a week’) (Jantzer et al. 2019). Additionally, the subscales for the five different types of bullying were also calculated. The FBS demonstrated high internal consistency with Cronbach's α = 0.87 (Shaw et al. 2013). Cronbach's α of the total score of the current study was 0.91, for the scale victimisation 0.94 and perpetration 0.80.

#### 2.2.2 Assessments of the Anorexia Registry Study, Completed Only By the AN Sample

##### 2.2.2.1 Eating Disorder Examination (EDE)

The German version of the semi‐structured Interview EDE (Hilbert et al. 2007) consists of 28 items that can be divided into four subscales (eating restraint, eating concerns, body shape concerns, weight concerns), of which 14 items are diagnostic items used to define ED diagnoses according to DSM‐IV. All items refer to the last 28 days and can be scored from 0 (item was not fulfilled) to 6 (item was fulfilled every day or to an extreme degree), a global score is the mean of the four subscales. The EDE has been shown to have good psychometric properties for all age groups with an internal consistency (Cronbach's *α*) global score of 0.88 (Hilbert et al. 2007).

##### 2.2.2.2 Body Mass Index (BMI) Percentiles and BMI Percentile *z*‐Scores

The severity of AN was additionally operationalised by age‐ and sex‐adjusted BMI percentiles, based on the World Health Organization (WHO) and Center for Disease Control and Prevention (CDC; Kuczmarski et al. 2002) growth standards. BMI percentiles are recommended when assessing weight in children and adolescents because they take developmental norms, gender and age into account (American Psychiatric Association 2013). In addition, the corresponding *z*‐scores from the BMI percentiles were used, as percentiles have limited utility when applied to cohorts that include participants with extreme BMI values (e.g., below the 1st percentile; Wang and Chen 2012). In a normal distribution, a *z*‐score indicates the distance in standard deviations (SDs) from a given value and a *z*‐score of + 1/−1 is one SD above/below the population mean (Martinez‐Millana et al. 2018).

### 2.3 Statistical Analyses

The samples include two clinical adolescent groups, one with the primary diagnosis of AN and another with a general psychiatric diagnosis excluding AN based on MINI‐KID results. Patients were matched for age, sex and global functioning (CGAS) using Coarsened Exact Matching (CEM). CEM is a common procedure to adjust targeted group sizes (i.e., AN) and allows for weighted matching (Blackwell et al. 2009). The global functioning is coarsened into 10 categories (1–10, 11–20, 21–30…, 91–100), age in years is converted to integers (11, 12…, 18), and gender is kept as it is (female, male), resulting in 160 strata. Means and SD were calculated for the descriptive statistics of the continuous variables. For the nominal variables, the exact numbers and proportions are reported.

In order to calculate potential differences in parental relationship between patients with AN and CC (H1), linear weighted regression analyses were calculated for the two groups as independent variables and the characteristics of the parental relationship as the dependent variables. These included the overall relationship quality as the main scale (BQG), parental relationship to mother (BQM) and father (BQF), parenting discrepancy as well as all subscales. Age and global functioning were used as control variables. The weights for each stratum were calculated using the CEM method. For all regression analyses, the distribution of the residuals was checked visually, showing no multicollinearity (VIF < 5) and no heteroscedasticity for any group (non‐significant Breusch‐Pagan test).

Weighted logistic regressions were performed to examine whether the two groups (AN and CC) differed in their experience of bullying (H2). The presence or absence of bullying was considered as the dependent variable and group as the independent variable. CEM weights were used, and analyses were controlled for age and general functioning, but not for sex as only female patients were recruited.

The outcome variable anorectic symptom severity was assessed separately by EDE global score, BMI percentiles and BMI percentile *z*‐scores. The association between parental relationship or peer bullying and symptom severity (H3, H4) were calculated by linear regression models with separate models for each parental relationship quality (maternal, paternal and parental discrepancy)/bullying (victimisation, perpetration) as the predictor variable. To test if parental relationship serves as a protective factor for the association between bullying and anorectic symptom severity, linear regression analyses with an interaction term between the parental relationship variables and bullying (yes/no) were conducted for the AN group (H5). Eight separate models were fitted for each outcome variable, that is a separate model for each combination of bullying (victimisation, perpetration) and parental relationship (BQG total score and the three subscales BQM, BQF, parental discrepancies). In total 24 interaction terms were tested. Global functioning was included as control variable in all models (H3, H4, H5) to account for its potential influence on the association. Age was included only for EDE, as the BMI percentile and *z*‐score include already a correction for age.

The effect size Cohen's *f* ^2^ was calculated for all linear regression analyses. Cohen's *f* ^2^ of 0.10 was interpreted as a small effect, 0.25 as a medium effect and 0.50 as a large effect (Cohen 1988). All *p*‐values were two‐sided and the alpha level was set at 0.05. Because of multiple testing we report the false discovery rate corrected *p*‐values for each hypothesis using the Benjamini‐Hochberg procedure (Benjamini and Hochberg 2000). Statistical analyses were performed using the statistical software STATA version 18 (StataCorp 2023).

## 3 Results

### 3.1 Participants

**FIGURE 1:**
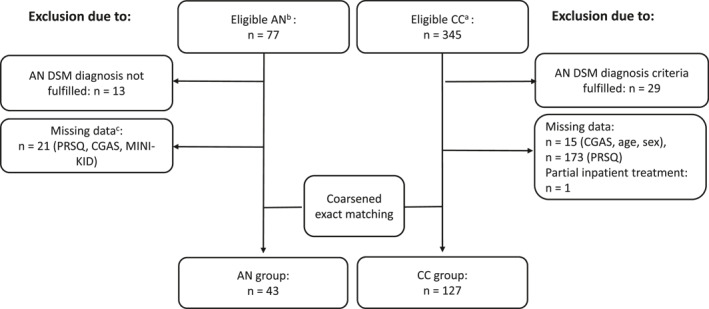
Flow chart of the inclusion and exclusion of participants for AN and CC groups. AN = anorexia nervosa sample; CC = clinical control group; CGAS = Children's Global Assessment Scale; MINI‐KID = Mini International Neuropsychiatric Interview for Children and Adolescents; PRSQ = parental representation screening questionnaire. ^a^ Eligible CC group: includes participants with data exclusively from the BeBaDoc study. ^b^ Eligible AN group: includes 56 participants with data from the Anorexia Registry Study and from the BeBaDoc study and a further 21 participants with data only from the Anorexia Registry Study. ^c^ 21 participants with data only from the Anorexia Registry Study were excluded because they had no data (e.g., PRSQ, CGAS, MINI‐KID) from the BeBaDoc study.

Combining the data from the two samples (BeBaDoc, *n* = 401, and the Anorexia Registry Study, *n* = 77) resulted in 345 participants with BeBaDoc data only, and 56 with data from both studies. Of the 345 participants with BeBaDoc data only, one was excluded due to day‐care treatment, 15 due to missing matching data (age, sex, CGAS), and 29 for meeting the MINI‐KID diagnostic criteria for AN, an exclusion criterion for the CC group. In addition, 13 of the 56 participants with data in both samples were excluded, because they did not meet the criteria for AN as assessed by the MINI‐KID (an inclusion criterion for the AN group). After performing the CEM, one matched CC was excluded due to missing PRSQ data. This resulted in a final sample of *N* = 170, including 127 CC, who were matched to 43 patients with AN. Of the 160 possible categories (10 CGAS, 2 sex, and 8 age categories), 19 strata were populated in both groups. A detailed overview is provided in Figure 1.

The mean age of the sample was 14.88 years (SD = 1.04; range 12–16 years), all participants were female. The CGAS range was 21–80, indicating severe to moderate impairment. The 43 participants with AN reported an EDE global score of 3.86 (SD = 1.05), a BMI between 11.70 and 19.10, BMI percentiles between < 1 and 36, and corresponding *z*‐scores between −5.76 and −0.35; these indices are consistent with previous literature for comparable samples (Gradl‐Dietsch et al. 2024; Hilbert and Tuschen‐Caffier 2016). In both samples, the majority of the patients suffered from the co‐occurrence of multiple psychiatric diagnoses such as affective or anxiety disorders (CC: 91.34%, AN: 88.37%). Demographic and clinical descriptive statistics for the total sample and for each group separately are presented in Table 1.

**TABLE 1:** Characteristics of the total sample and by clinical status (AN and CC).

Characteristics	Total sample (*n* = 170)	AN (*n* = 43)	CC (*n* = 127)
Age; mean (SD)	14.88 (1.04)	14.60 (1.03)	14.97 (1.04)
CGAS; mean (SD)	49.79 (11.58)	51.60 (13.05)	49.17 (11.03)
Level of education; *n* (%)^a^			
Primary school (ISCED levels 0−1; at least 6 school years)	19 (11.2)	3 (7.0)	16 (12.60)
Secondary school (ISCED level 2; 9−10 school years)	115 (67.6)	30 (69.8)	85 (66.9)
High school (ISCED level 3; 12−13 school years)	35 (20.6)	10 (23.3)	25 (19.7)
Other	1 (0.6)	0	1 (0.8)
ED diagnoses (MINI‐KID); *n* (%)			
Bulimia nervosa	16 (9.4)	0	16 (12.6)
Anorexia nervosa diagnoses			
AN restrictive type	42 (24.7)	42 (97.7)	0
AN binge‐purging type	1 (0.6)	1 (2.3)	0
Other (comorbid) psychiatric diagnoses (MINI‐KID); *n* (%)			
Any affective disorder	130 (76.47)	33 (76.74)	97 (76.38)
Any anxiety disorder	133 (78.24)	25 (58.14)	108 (85.04)
Any obsessive‐compulsive disorder	40 (23.53)	7 (16.28)	33 (25.98)
Any posttraumatic stress disorder	33 (19.41)	5 (11.63)	28 (22.05)
Any substance use disorder	43 (25.29)	1 (2.33)	42 (33.07)
Any behavioural and emotional disorders	40 (23.53)	2 (4.65)	38 (29.92)
Any psychotic disorder	34 (20.00)	7 (16.28)	27 (21.26)
EDE global score; mean (SD)	—	3.86 (1.05)	—
Duration of AN in months; mean (SD)	—	14.51 (13.39)	—
BMI; mean (SD)	—	14.96 (1.49)	—
BMI percentiles; mean (SD)	—	3.09 (6.17)	—
BMI percentiles *z*‐score; mean (SD)	—	−2.79 (1.33)	—

Abbreviation: AN = anorexia nervosa group; BMI = body mass index. CC = clinical control group; CGAS = Children's Global Assessment Scale; EDE = Eating Disorder Examination; ISCED = International Standard Classification of Education; MINI‐KID = Mini International Neuropsychiatric Interview for Children and Adolescents;

### 3.2 Parental Relationship in Patients With AN and CC

Weighted regression analyses used to examine group differences between AN and CC, revealed several differences in parental relationships (see Table 2 and Supporting Information S1: eTable 2). Patients with AN reported an overall more positive relationship with their parents (BQG; corrected *p*‐value = 0.001, Cohen's *f* ^2^ = 0.10) as well as individually with their mothers (BQM; corrected *p*‐value = 0.028, Cohen's *f* ^2^ = 0.04) and fathers (BQF; corrected *p*‐value = 0.014, Cohen's *f* ^2^ = 0.05) compared to CC. Additionally, AN patients reported a lower average of parental discrepancy compared to CC (*p‐*value = 0.005, Cohen's *f* ^2^ = 0.07). Descriptive data on the relationship quality subscales between the two patient groups (AN, CC) are presented in Supporting Information S1: eTable 1.

**TABLE 2:** Group differences in parental relationship between patients with AN and CC.

			95% CI				
PRSQ	Group differences *B*	*SE*	*LL*	*UL*	*p*	*p adj*.	*f* ^2^
BQG	18.34	4.54	9.37	27.30	0.000	0.002**	0.10
BQM	5.37	2.10	1.23	9.51	0.011	0.079	0.04
BQF	5.30	1.76	1.82	8.78	0.003	0.028*	0.05
Parental discrepancies	−7.67	2.26	12.14	−3.20	0.001	0.013*	0.07
Cohesion‐M	5.41	1.99	1.48	9.34	0.007	0.027*	0.04
Identification‐M	4.79	1.90	1.04	8.53	0.013	0.025*	0.04
Autonomy‐M	5.29	2.01	1.32	9.26	0.009	0.025*	0.04
Conflict‐M	−4.00	2.09	−8.13	0.14	0.058	0.098	0.02
Punishment‐M	−2.23	1.34	−4.89	0.42	0.098	0.154	0.02
Rejection‐M	−4.28	1.62	−7.47	−1.09	0.009	0.028*	0.04
Emotional boundary overstepping‐M	1.60	1.77	−1.89	5.09	0.366	0.474	0.00
Anxiety‐M	−0.37	1.78	−3.87	3.14	0.836	0.876	0.00
Help‐M	−1.37	2.14	−5.59	2.85	0.523	0.639	0.00
Cohesion‐F	8.33	1.98	4.43	12.24	0.000	0.001*	0.11
Identification‐F	7.17	1.99	3.23	11.10	0.000	0.003*	0.08
Autonomy‐F	5.01	2.36	0.34	9.67	0.036	0.065	0.03
Conflict‐F	−0.17	2.30	−4.72	4.38	0.941	0.941	0.00
Punishment‐F	−0.65	1.29	−3.20	1.89	0.613	0.674	0.00
Rejection‐F	−2.56	1.66	−5.84	0.71	0.124	0.182	0.01
Emotional boundary overstepping‐F	3.72	1.45	0.85	6.59	0.011	0.025*	0.04
Anxiety‐F	1.17	1.92	−2.62	4.95	0.543	0.629	0.00
Help‐F	−1.61	1.77	−5.11	1.88	0.364	0.500	0.00

Abbreviations: BQG = overall parental relationship quality; BQM = relationship quality to the mother; BQF = relationship quality to the father; F = father. M = mother; PRSQ = parental representation screening questionnaire.

*p < 0.05.

A more detailed examination of the subscales revealed that patients with AN reported significantly more cohesion (M: corrected *p‐*value = 0.027, Cohen's *f* ^2^ = 0.04; F: corrected *p‐*value = 0.001, Cohen's *f* ^2^ = 0.11) and identification (M: corrected *p‐*value = 0.025, Cohen's *f* ^2^ = 0.04; F: corrected *p‐*value = 0.003, Cohen's *f* ^2^ = 0.08) in relationships to both mothers and fathers, less rejection (corrected *p‐*value = 0.028, Cohen's *f* ^2^ = 0.04) and more autonomy (corrected *p‐*value = 0.025, Cohen's *f* ^2^ = 0.04) in maternal relationships, as well as more emotional boundary overstepping in paternal relationships (corrected *p‐*value = 0.025, Cohen's *f* ^2^ = 0.04) than CC.

### 3.3 Peer Relationships in Patients With AN and CC

Descriptive statistics (see Table 3 and Supporting Information S1: eTable 3) indicated that 51.2% of patients with AN and 72.4% of CC reported having been victims of bullying. Within the AN group, 11.6% reported having experienced frequent bullying (once a week or more) compared to 31.5% in the CC group. In addition, the results show that only 18.6% of individuals in the AN group reported having been perpetrators of bullying compared to 47.2% in the CC group.

**TABLE 3:** Descriptives of peer relationships between AN and CC patients.

Characteristics FBS (*n*; %)	Total (*n* = 170)	AN (*n* = 43)	CC (*n* = 127)
Victimisation (yes/no)			
Yes	114 (67.1)	22 (51.2)	92 (72.4)
Perpetration (yes/no)			
Yes	68 (40.0)	8 (18.6)	60 (47.2)

Abbreviations: AN = anorexia nervosa sample; CC = clinical control group; FBS = forms of bullying scale.

Examining bullying, the AN group were significantly less likely to report bullying victimisation (OR = 0.43, corrected *p*‐value 0.030) and less likely to engage in bullying perpetration (OR = 0.26, corrected *p*‐value 0.006) than CC (see Table 4 and Supporting Information S1: eTable 4).

**TABLE 4:** Group differences in victimisation and perpetration between patients with AN and CC.

FBS	Group differences *Odds ratio*	*SE*	95% CI	*p*	*p adj*.	
			*LL UL*			*f* ^2^
Victimisation	0.43	0.17	0.20 0.92	0.030	0.030*	0.43
Perpetration	0.26	0.12	0.10 0.63	0.003	0.006*	0.26

Abbreviation: FBS = forms of bullying scale.

*p < 0.05.

### 3.4 Association Between Parental Relationship or Bullying and Severity of AN

Among patients with AN, there was no evidence of an association between parental relationship or bullying and anorexic severity indices (see Table 5 and Supporting Information S1: eTable 5).

**TABLE 5:** Association between symptom severity and parental relationship/bullying in patients with AN.

Outcome	Predictor	Coefficient	SE	95% CI		*p*	*p*	*f* ^2^
		B		LL	UL		*adj.*	
EDE	BQG	0.00	0.01	−0.01	0.01	0.834	0.945	0.00
	BQM	0.00	0.02	−0.03	0.03	0.987	0.987	0.00
	BQF	0.00	0.02	−0.03	0.04	0.840	0.945	0.00
	Parental discrepancies	−000	0.01	−0.03	0.02	0.803	0.945	0.00
	Victimisation	−0.30	0.35	−1.01	0.42	0.402	0.945	0.02
	Perpetration	0.25	0.47	−0.70	1.20	0.596	0.945	0.01
BMI P	BQG	0.03	0.04	−0.05	0.10	0.487	0.945	0.01
	BQM	0.01	0.10	−0.19	0.21	0.943	0.987	0.00
	BQF	0.06	0.10	−0.14	0.25	0.565	0.945	0.01
	Parental discrepancies	−0.06	0.07	−0.21	0.08	0.391	0.945	0.02
	Victimisation	−2.60	1.93	−6.49	1.29	0.184	0.945	0.05
	Perpetration	1.19	2.44	−3.75	6.13	0.629	0.945	0.01
BMI P *z*	BQG	000	0.01	−0.01	0.02	0.562	0.945	0.01
	BQM	−000	0.02	−0.05	0.04	0.819	0.945	0.00
	BQF	0.01	0.02	−0.04	0.05	0.806	0.945	0.00
	Parental discrepancies	−0.02	0.02	−0.05	0.01	0.269	0.945	0.03
	Victimisation	0.13	0.42	−0.72	0.98	0.759	0.945	0.00
	Perpetration	0.90	0.51	−0.12	1.93	0.083	0.945	0.08

Abbreviations: *n* = 43; EDE = Eating Disorder Examination; BMI P = Body Mass Index percentiles; BMI P z = Body Mass Index percentile *z*‐scores; BQG = overall parental relationship quality; BQM = relationship quality to the mother; BQF = relationship quality to the father; B = unstandardised regression coefficient; B SE = standard error of B; LL = lower level; UL = upper level.

*p < 0.05.

### 3.5 Influence of Parental Relationships on the Association Between Bullying and Severity of AN

None of the parental relationship variables moderated the association between bullying and indices of AN severity (see Table 6 and Supporting Information S1: eTable 6). Uncorrected *p*‐values indicates some significant associations, but they did not withstand *p*‐value correction and were most likely due to chance.

**TABLE 6:** Results of multiple regression analyses in patients with AN: Prediction of AN severity by bullying with parental relationship as moderator.

Outcome					95% CI				
	Predictor	Moderator	Coefficient *B*	SE	LL	UL	*p*	*p adj*.	*f* ^2^
EDE	Victimisation	BQG	−000	0.01	−0.03	0.02	0.747	0.927	0.00
BMI P			−0.09	0.07	−0.24	0.05	0.207	0.827	0.04
BMI P z			−0.04	0.002	−0.07	−0.01	0.014*	0.249	0.17
EDE	Perpetration		0.01	0.02	−0.03	0.04	0.753	0.927	0.00
BMI P			0.05	0.10	−0.14	0.25	0.588	0.927	0.01
BMI P z			0.	0.02	−0.04	0.04	0.900	0.927	0.00
EDE	Victimisation	BQM	−0.04	0.04	−0.11	0.04	0.333	0.927	0.03
BMI P			−0.12	0.21	−0.55	0.31	0.574	0.927	0.01
BMI P z			−0.08	0.05	−0.17	0.01	0.082	0.652	0.08
EDE	Perpetration		0.01	0.06	−0.11	0.12	0.927	0.927	0.00
BMI P			0.17	0.29	−0.43	0.76	0.573	0.927	0.01
BMI P z			0.001	0.06	−0.12	0.13	0.913	0.927	0.00
EDE	Victimisation	BQF	−0.02	0.03	−0.09	0.05	0.581	0.927	0.01
BMI P			−0.16	0.19	−0.53	0.22	0.408	1.	0.02
BMI P z			−0.06	0.04	−0.14	0.02	0.119	0.715	0.07
EDE	Perpetration		−0.01	0.04	−0.09	0.06	0.745	0.927	0.00
BMI P			0.06	0.21	−0.36	0.47	0.790	0.927	0.00
BMI P z			−0.01	0.04	−0.08	0.09	0.865	0.927	0.00
EDE	Victimisation	Parental discre‐pancies	−0.01	0.03	−0.07	0.04	0.644	0.927	0.01
BMI P			0.21	0.14	−0.08	0.50	0.156	0.747	0.06
BMI P z			0.07	0.03	0.01	0.13	0.021*	0.249	0.15
EDE	Perpetration		−0.05	0.04	−0.14	0.04	0.244	0.838	0.04
BMI P			−0.16	0.25	−0.66	0.34	0.524	0.927	0.01
BMI P z			−0.01	0.05	−0.11	0.09	0.831	0.927	0.00

Abbreviations: *n* = 43; EDE = Eating Disorder Examination; BMI P = Body Mass Index percentiles; BMI P z = Body Mass Index percentile *z*‐scores; BQG = overall parental relationship quality; BQM = relationship quality to the mother; BQF = relationship quality to the father; B = unstandardised regression coefficient; B SE = standard error of B; LL = lower level; UL = upper level.

^*^ *p* < 0.05.

## 4 Discussion

The objective of this cross‐sectional study was to compare perceived relationship quality with parents and bullying between adolescent inpatients with AN and CC. In addition, the study examined the associations between relationship characteristics and symptom severity in patients with AN. The association between bullying and symptom severity was also investigated, assuming that the parental relationship buffers this association.

This study yielded several key findings. First, adolescents with AN reported a more positive relationship quality with their parents than adolescents in the CC group. They experienced better relationships with both mothers and fathers, and lower levels of parental discrepancy. Second, patients with AN exhibited a lower prevalence of bullying victimisation and perpetration compared to the CC group. Third, after adjusting for family error rates, no significant correlations were found between the parental relationships or bullying with AN severity, nor did parental relationship moderate the association between bullying experiences and AN severity.

The hypothesis that patients with AN would report fewer difficulties in their parental relationships than CC was confirmed for the general parental relationship as well as for the parental discrepancies. This finding partially aligns with previous research comparing AN patient groups with healthy adolescents (Gruber et al. 2020; Tetley et al. 2014; Vidović et al. 2005), but contradicts earlier studies suggesting similar levels of parental relationship difficulties between AN and CC (Tetley et al. 2014). The divergent outcomes may be attributable to the varying operationalisation of relationships or the particular composition of diagnoses within the samples. Moreover, studies have indicated the presence of clinical distinctions between patients with AN and other eating disorders (Tetley et al. 2014; Vidović et al. 2005; Yamamiya and Stice 2024), with symptoms of eating disorders also frequently observed as comorbidity in clinical samples of adolescents (Johnson et al. 2002; Lee and Vaillancourt 2024). Our findings extend current literature by demonstrating that AN patients report fewer difficulties in both parental relationships and discrepancies when compared to a CC with various psychiatric disorders. Longitudinal findings may help explain this effect, suggesting that parent‐child relationships were resourceful prior to the onset of anorexia nervosa and became more conflictual as a consequence of the disorder (Korotana et al. 2018).

Further examination of the parental relationship subscales in the current study, separately for mothers and fathers, showed that cohesion, identification and autonomy may act as maternal resources while rejection may be a risk factor. For father relationships, cohesion and identification were important resources while emotional boundary overstepping appears to be a relevant risk factor. These findings are consistent with previous findings on parents' interpersonal style, which appears to be less caring, highly disengaged, less interwoven, rigid and associated with less cohesion and communication between patients with AN and other ED (Tafà et al. 2017; Treasure et al. 2020). In particular, previous research has identified restrictions of behavioural autonomy (Laporte and Guttman 2007; Tetley et al. 2014), contradictory relationship styles between parents of AN patients, such as lower care versus higher protection (Tetley et al. 2014) and contradictory educational behaviour, such as rejection versus overprotection (Gruber et al. 2020). Evidently, different resources and risk factors are associated with both parents during the sensitive phase of adolescence. This can be explained by the developmental tasks of adolescence, such as emotion regulation, self‐acceptance or separating from primary reference persons and turning to peers. During this complex intrapsychic process, contradictory behaviours on the part of one or both parents can contribute to the maintenance of AN (Gander et al. 2015; Korotana et al. 2018). Consequently, families with inconsistent parenting styles and approaches to AN education may experience greater conflict, avoidance, or overprotection, which can hinder adolescents' development of autonomy and adversely affect the course of the illness or overall development (Cerniglia et al. 2017; Gruber et al. 2020; Korotana et al. 2018).

The second hypothesis, that individuals with AN report a lower prevalence of bullying than the CC group was confirmed. Patients with AN reported suffering from victimisation substantially, but to a lesser extent than CC. This correlation confirms the numerous and very clear findings to date, according to which victimisation is a risk factor for the development of an ED (Breton et al. 2023; Lee and Vaillancourt 2024; Lie et al. 2019). In view of the paucity of previous studies comparing with CC, and given that these studies have also found differences between the AN and CC groups (Mayes et al. 2015), our results extend these findings.

No association was identified between the quality of parental relationships or bullying with the severity of symptoms in individuals with AN. This suggests that relationship quality may not be directly related to AN severity. Balottin et al. (2018) also failed to identify an association between parental relationship quality and AN severity, although a more detailed analysis revealed an association between the triadic family system and symptom severity. In terms of peer relationship, bullying victimisation was more prevalent among patients with ED but there was no correlation between its prevalence and symptom severity in previous research (Lie et al. 2019, 2021). The absence of substantial correlations with symptom severity can be ascribed, firstly, to the limited sample size and, secondly, to the marked severity of underweight symptoms within the sample, as demonstrated by the skewed distribution of the data. On the one hand, this results in a highly selective group based on strict criteria for underweight, and, on the other hand, to people who are consistently exposed to high life stress. This leads to the question of whether their response behaviour is one‐dimensional due to their underweight, to their cognitive capability of answering the questionnaires in a differentiated manner or to social accepted response tendencies.

It is worth noting that the current study used a number of criteria to assess AN severity, namely general ED symptoms (EDE) and two weight measures, which were differentially associated to parental relationships and bullying experiences. This suggests that interview and weight measures may be measuring different elements of AN. As mentioned above, weight measures in the present study are limited by the skewed distribution of the percentile values, minimal variance in severity and compressed *z*‐scores (Wang and Chen 2012). Interestingly, other studies have also demonstrated a lack of correlation between EDE and weight measures (Kemp et al. 2023; Toppino et al. 2022; Weigel et al. 2016).

The fifth hypothesis, that parental relationship may moderate the association between victimisation and AN severity was not confirmed. The protective or risk‐promoting influence of parents on peer‐relationships has been documented in several studies, but with various methodological approaches (Gander et al. 2015; Holtom‐Viesel and Allan 2014; Schulz et al. 2023; Tetley et al. 2014). Conversely, Mikhaylova and Bochaver (2022) showed that a hostile parental relationship style increases the risk of victimisation at school in patients with AN. Yamamiya and Stice (2024) demonstrated that stressful ED‐related interactions to parents or peers reinforce symptom severity in a large prospective school cohort. The present finding contradicts these results, as the association between relationship qualities and AN severity could not be verified. One potential explanation is that the level of victimisation in our study was insufficient for parental buffering function to be statistically detectable. Alternatively, the range of symptom severity within the present sample may has been too narrow to identify any significant effects. This suggests that the severity of AN may not be primarily explained by relational factors, but rather by other maintaining mechanisms ‐ particularly in severely affected inpatients. However, the findings support the hypothesis, that patients with AN tend to experience more resourceful relationships, which then deteriorate bidirectionally during the acute phase of the illness.

## 5 Clinical Implications

Current treatment guidelines for eating disorders recommend consistent assessment of parental relationships and integration of family therapy (e.g., FBT, Ciao et al. 2015; Wallis et al. 2017). These family‐focused approaches should address the relationship with both parents, as our study showed discrepant interactions with mother and father, rather than focussing on a single parent, and aim to resolve maladaptive discrepancies in parenting by means of coherent education. Therefore, interventions are encouraged to enhance self‐esteem, insight into interpersonal problems, social skills training (e.g., dealing with rejection or emotional overstepping), and a supportive atmosphere. Strengthening parental relationships provides a critical foundation for treatment progress, as AN is an ambiguous illness that often impedes therapeutic progress through self‐perpetuating and challenging interpersonal patterns.

## 6 Strengths and Limitation

To our knowledge, this is the first study to examine the perceived quality of relationships with the mother and the father and bullying in adolescent inpatients with AN compared with CC and their association with AN severity. A notable strength of this study is the use of validated semi‐structured interviews in a highly specialised domain of research, where only limited studies have been conducted. Moreover, unlike traditional 1:1 matching procedures with the AN sample, the CEM method ensured comparability in clinical severity between samples while allowing for the inclusion of a larger pool of clinical controls in the subsequent regression analyses. However, we only measured the severity of AN and the relationship quality with parents and bullying at baseline, and did not conduct additional assessments during or at the end of treatment. This prevented us from examining the impact of relationship quality on treatment outcome. Second, the generalisability of the findings is limited due to small and female samples. Third, given that both patient groups exhibited a high level of general symptom severity, an additional comparison with a wider distribution of AN severity would probably yield more distinct results regarding the impact of relationship quality on symptom severity and vice versa. Forth, significant external social influences on the parent‐child relationship were not considered in the analyses, including isolation or concerns during the Covid pandemic, or particular generational characteristics. Finally, given the limited number of studies about parental relationship in CC groups, the prediction of the group comparison between AN and CC was unclear, which would also have allowed a non‐directional hypothesis.

## 7 Conclusion and Future Directions

Patients with AN perceived their relationships with parents more positively to CC, and peer victimisation seemed less prevalent in the AN group. Future studies with larger sample sizes and a longitudinal design should examine the association between relationship quality (with parents and peers) and anorectic pathology throughout the course of treatment. Additionally, comparisons with a sample with a greater distribution of symptom severities may be conducted to identify the resources and risk factors for developing anorexia nervosa. Further developments in this field could involve the simultaneous analysis of parents', peers', and therapists' perceptions of relationship quality, on the one hand, and the operationalisation of relationship quality based on interaction analyses, on the other.

## Author Contributions

**Armita Tschitsaz:** conceptualization, writing – original draft, investigation, project administration, data curation, formal analysis, writing – review and editing. **Andrea M. Schumacher:** writing – review and editing, investigation, data curation. **Stefan Lerch:** formal analysis, data curation, writing – review and editing. **Andrea Wyssen:** writing – review and editing. **Ines Mürner‐ Lavanchy:** investigation, writing– review and editing. **Julian Koenig:** writing – review and editing. **Marialuisa Cavelti:** writing – review and editing. **Michael Kaess:** conceptualization, funding acquisition, supervision, writing – review and editing. **Franziska Schlensog‐Schuster:** supervision, writing – review and editing.

## Funding

The costs of this study were covered by department funds of the University Department of Child and Adolescents Psychiatry and Psychotherapy at the University of Bern (responsible: Prof. Michael Kaess). Marialuisa Cavelti was supported by a grant from the Swiss National Science Foundation (PZ00P1_193279).

## Ethics Statement

The present study was approved by the Swiss cantonal ethics committees of Bern (BeBaDoc Ethics ID: 2018‐01339; and Anorexia Registry Ethics ID: 2021‐00234). Prior to participation, written informed consent was obtained from all participants (and their parents/legal custodians for participants under 14 years of age) after a comprehensive explanation of the study procedures.

## Consent

The authors have nothing to report.

## Conflicts of Interest

The authors declare no conflicts of interest.

## Supporting information


Supporting Information S1

## Data Availability

The data that support the findings of this study are available from the corresponding author upon reasonable request.
