# Supplementary material for: Parental and Peer Relationships and Their Impact on Symptom Severity in Adolescent Patients With Anorexia Nervosa
Source: Eur Eat Disord Rev. 2025 Dec 27;34(3):845–57. doi: 10.1002/erv.70072 (PMC13048737; doi:10.1002/erv.70072)
Supplement: Supplementary file 1 — Supporting Information S1 [file ERV-34-845-s001.docx]

**Supporting Information for**

***“*Parental and peer relationships and their impact on symptom severity in adolescent patients with anorexia nervosa*”***

DOI: 10.1002/erv.70072

**Contents:** 6 eTables, standard MS office format (Word)

- **S1 eTable 1**. *Descriptives of* *parental relationship subscales between AN and CC patients*
- **S1 eTable 2**. *Overall model fit of group differences in parental relationships AN patients and CC*
- **S1 eTable 3**. *Descriptives of* *peer relationships between AN and CC patients*
- **S1 eTable 4**. *Overall model fit of group differences in victimization and perpetration between patients with AN and CC*
- **S1 eTable 5.**  *Overall model fit of the association between symptom severity and parental / peer relationship in patients with AN*
- **S1 eTable 6.** *Overall model fit of multiple regression analyses in patients with AN: Prediction of AN severity by peer relationship with parental relationship as moderator*

**eTable 1**

*Descriptives of* *parental relationship subscales between AN and CC patients*

| Subscales PRSQ; mean (*SD*) | Total  (n = 170) | AN  (n = 43) | CC  (n = 127) |
| --- | --- | --- | --- |
| BQG | 44.45 (27.17) | 59.09 (28.61) | 39.50 (24.89) |
| BQM | 48.42 (11.61) | 52.51 (10.26) | 47.04 (11.75) |
| BQF | 48.58 (10.97) | 53.63 (11.97) | 46.87 (10.11) |
| Parental discrepancies | 52.55 (13.00) | 47.05 (13.65) | 54.41 (12.28) |
| Cohesion-M | 47.70 (11.88) | 52.09 (9.81) | 46.21 (12.18) |
| Identification-M | 48.49 (11.26) | 52.49 (7.90) | 47.13 (11.92) |
| Autonomy-M | 49.81 (11.36) | 54.00 (9.11) | 48.39 (11.72) |
| Conflict-M | 48.89 (11.64) | 46.47 (9.93) | 49.72 (12.10) |
| Punishment-M | 48.44 (7.67) | 46.63 (5.84) | 49.05 (8.13) |
| Rejection-M | 52.62 (9.30) | 49.56 (7.40) | 53.66 (9.67) |
| Emotional boundary overstepping-M | 50.12 (10.30) | 51.91 (8.72) | 49.51 (10.74) |
| Anxiety-M | 49.95 (10.07) | 49.88 (7.40) | 49.97 (10.86) |
| Help-M | 47.43 (12.05) | 46.65 (11.03) | 47.69 (12.41) |
| Cohesion-F | 46.63 (11.84) | 53.26 (11.83) | 44.39 (11.01) |
| Identification-F | 48.43 (11.56) | 54.26 (9.63) | 46.46 (11.52) |
| Autonomy-F | 49.05 (13.33) | 54.02 (9.79) | 47.36 (13.97) |
| Conflict-F | 48.96 (13.34) | 47.74 (11.27) | 49.37 (13.99) |
| Punishment-F | 48.73 (8.01) | 47.30 (7.58) | 49.21 (8.12) |
| Rejection-F | 53.64 (9.98) | 51.12 (8.62) | 54.49 (10.30) |
| Emotional boundary overstepping-F | 48.87 (8.60) | 51.74 (8.86) | 47.90 (8.33) |
| Anxiety-F | 48.13 (11.25) | 49.05 (9.66) | 47.82 (11.76) |
| Help-F | 46.18 (10.02) | 44.84 (8.36) | 46.63 (10.52) |

*Note.* AN = Anorexia nervosa sample, CC = Clinical control group, PRSQ = Parental Representation Screening Questionnaire, BQG = overall parental relationship quality, BQM = relationship quality to the mother, BQF = relationship quality to the father, M = mother, F = father.

**eTable 2**

*Overall model fit of group differences in parental relationships between AN patients and CC*

| Model summary PRSQ  (n = 170) | *F*(3, 166) | *p* |  | | *R^2^ adj.* |
| --- | --- | --- | --- | --- | --- |
| BQG | 8.13 | <.001 | | *** | 0.112 |
| BQM | 3.13 | .027 | | * | 0.036 |
| BQF | 7.89 | <.001 | | *** | 0.109 |
| Parental discrepancies | 4.35 | .006 | | ** | 0.056 |
| Cohesion-M | 4.18 | .007 | | ** | 0.053 |
| Identification-M | 2.68 | .049 | |  | 0.029 |
| Autonomy-M | 3.51 | .017 | | * | 0.043 |
| Conflict-M | 1.40 | .246 | |  | 0.007 |
| Punishment-M | 2.33 | .076 | |  | 0.023 |
| Rejection-M | 2.94 | .035 | |  | 0.033 |
| Emotional boundary overstepping-M | 0.49 | .691 | |  | -0.009 |
| Anxiety-M | 1.59 | .193 | |  | 0.010 |
| Help-M | 0.43 | .733 | |  | -0.010 |
| Cohesion-F | 11.08 | <.001 | | *** | 0.152 |
| Identification-F | 5.44 | .001 | | *** | 0.073 |
| Autonomy-F | 3.84 | .011 | | * | 0.048 |
| Conflict-F | 1.69 | .172 | |  | 0.012 |
| Punishment-F | 4.85 | .003 | | ** | 0.064 |
| Rejection-F | 1.95 | .123 | |  | 0.017 |
| Emotional boundary overstepping-F | 3.14 | .027 | | * | 0.037 |
| Anxiety-F | 0.92 | .430 | |  | -0.001 |
| Help-F | 0.41 | .747 | |  | -0.011 |

*Note.* PRSQ = Parental Representation Screening Questionnaire, BQG = overall parental relationship quality, BQM = relationship quality to the mother, BQF = relationship quality to the father, M = mother, F = father. * *p* < .05 ** *p* < .01 *** *p* < .001

**eTable 3**

*Descriptives of* *peer relationships between AN and CC patients*

| Characteristics FBS (n; %) | Total  (n = 170) | AN  (n = 43) | CC  (n = 127) |
| --- | --- | --- | --- |
| Frequency of victimization |  |  |  |
| never | 56 (32.9) | 21 (48.8) | 35 (27.6) |
| one or two times | 33 (19.4) | 11 (25.6) | 22 (17.3) |
| every few weeks | 36 (21.2) | 6 (14.0) | 30 (23.6) |
| once a week | 12 (7.1) | 1 (2.3) | 11 (8.7) |
| several times a week or more | 33 (19.4) | 4 (9.3) | 29 (22.8) |
|  |  |  |  |
| Frequency of perpetration |  |  |  |
| never | 102 (60.0) | 35 (81.4) | 67 (52.8) |
| one or two times | 37 (21.8) | 3 (7.0) | 34 (26.8) |
| every few weeks | 24 (14.1) | 4 (9.3) | 20 (15.7) |
| once a week | 4 (2.4) | 1 (2.3) | 3 (2.4) |
| several times a week or more | 3 (1.8) | 0 (0.0) | 3 (2.4) |

*Note.* AN = Anorexia nervosa sample, CC = Clinical control group, *FBS* = Forms of Bullying Scale.

**eTable 4**

*Overall model fit of group differences in victimization and perpetration between patients with AN and CC*

| Model summary FBS (n = 170) | Χ^2^(3) | *p* | *McFadden R^2^* | |
| --- | --- | --- | --- | --- |
| Victimisation | 24.85 | <.001*** | | 0.112 |
| Perpetration | 26.70 | <.001*** | | 0.119 |

*Note. FBS* = Forms of Bullying Scale. * *p* < .05 ** *p* < .01 *** *p* < .001

**eTable 5**

*Overall model fit of the association between symptom severity and parental / peer relationship in patients with AN*

| Model summary (n = 43) | |  |  |  |
| --- | --- | --- | --- | --- |
| Outcome | Predictor | *F*(df between, df within) | *p* | *R^2^ adj.* |
| EDE |  | *F* (3, 39) |  |  |
|  | BQG | 0.10 | .959 | -0.069 |
|  | BQM | 0.09 | .966 | -0.070 |
|  | BQF | 0.10 | .959 | -0.069 |
|  | Parental discrepancies | 0.11 | .955 | -0.068 |
|  | Victimisation | 0.33 | .805 | -0.050 |
|  | Perpetration | 0.18 | .907 | -0.062 |
| BMI P |  | *F* (2, 40) |  |  |
|  | BQG | 0.70 | .501 | -0.014 |
|  | BQM | 0.45 | .638 | -0.027 |
|  | BQF | 0.62 | .541 | -0.018 |
|  | Parental discrepancies | 0.84 | .441 | -0.008 |
|  | Victimisation | 1.38 | .262 | 0.018 |
|  | Perpetration | 0.57 | .569 | -0.021 |
| BMI P z |  | F(2, 40) |  |  |
|  | BQG | 0.83 | .443 | -0.008 |
|  | BQM | 0.68 | .511 | -0.015 |
|  | BQF | 0.69 | .509 | -0.015 |
|  | Parental discrepancies | 1.30 | .283 | 0.014 |
|  | Victimisation | 0.70 | .501 | -0.014 |
|  | Perpetration | 2.29 | .115 | 0.058 |

*Note.* df = Degrees of freedom, BQG = overall parental relationship quality, BQM = relationship quality to the mother, BQF = relationship quality to the father, EDE = Eating Disorder Examination, BMI P= Body Mass Index percentiles, BMI P z = Body Mass Index percentile z-scores. * *p* < .05 ** *p* < .01 *** *p* < .001

**eTable 6**

*Overall model fit of multiple regression analyses in patients with AN: Prediction of AN severity by peer relationship with parental relationship as moderator*

| Model summary (n = 43) | | |  |  |  |
| --- | --- | --- | --- | --- | --- |
| Outcome | Predictor | Moderator | F (4, 38) | *p* | *R^2^ adj.* |
| EDE | Victimisation | BQG | 0.21 | .956 | -0.104 |
| BMI P |  |  | 1.19 | .331 | 0.018 |
| BMI P z |  |  | 2.16 | .092 | 0.100 |
| EDE | Perpetration |  | 0.15 | .979 | -0.113 |
| BMI P |  |  | 0.50 | .732 | -0.050 |
| BMI P z |  |  | 1.32 | .282 | 0.029 |
| EDE | Victimisation | BQM | 0.39 | .853 | -0.078 |
| BMI P |  |  | 0.75 | .562 | -0.024 |
| BMI P z |  |  | 1.17 | .339 | 0.016 |
| EDE | Perpetration |  | 0.11 | .9990 | -0.119 |
| BMI P |  |  | 0.36 | .836 | -0.065 |
| BMI P z |  |  | 1.09 | .376 | 0.008 |
| EDE | Victimisation | BQF | 0.25 | .937 | -0.098 |
| BMI P |  |  | 0.86 | .497 | -0.014 |
| BMI P z |  |  | 0.41 | .097 | 0.002 |
| EDE | Perpetration |  | 0.14 | .981 | -0.114 |
| BMI P |  |  | 0.40 | .806 | -0.060 |
| BMI P z |  |  | 0.34 | .111 | 0.017 |
| EDE | Victimisation | Parental discrepancies | 0.25 | .936 | -0.098 |
| BMI P |  |  | 1.56 | .206 | 0.050 |
| BMI P z |  |  | 2.17 | .090 | 0.101 |
| EDE | Perpetration |  | 0.42 | .831 | -0.074 |
| BMI P |  |  | 0.59 | .673 | -0.041 |
| BMI P z |  |  | 1.59 | .197 | 0.053 |

*Note.* BQG = overall parental relationship quality, BQM = relationship quality to the mother, BQF = relationship quality to the father, EDE = Eating Disorder Examination, BMI P= Body Mass Index percentiles, BMI P z = Body Mass Index percentile z-scores. * *p* < .05 ** *p* < .01 *** *p* < .001
